# Atrial and ventricular strain changes after transcatheter pulmonary valve replacement in patients with repaired tetralogy of Fallot: a feature tracking cardiac MRI study

**DOI:** 10.1007/s11604-025-01765-x

**Published:** 2025-03-12

**Authors:** Sercin Ozkok, Hatice Ozge Ciftci, Ilker Kemal Yucel, Dursun Muhammed Ozdemir, Kevser Banu Kose, Ahmet Celebi, Kerem Pekkan

**Affiliations:** 1 Department of Radiology, Basaksehir Cam and Sakura City Hospital, Istanbul, Turkey; 2 Department of Biomedical Engineering, Koç University, Istanbul, Turkey; 3 Department of Radiology, Dr. Ilhan Varank Sancaktepe Training and Research Hospital, Istanbul, Turkey; 4 Cerrahpasa Medical Faculty, Department of Pediatric Cardiology, Istanbul University-Cerrahpasa, Istanbul, Turkey; 5 Department of Pediatric Cardiology, Dr. Siyami Ersek Thoracic and Cardiovascular Surgery Training and Research Hospital, Istanbul, Turkey; 6 Department of Biomedical Engineering, Istanbul Medipol University, Istanbul, Turkey; 7 Department of Mechanical Engineering, Koc University, Istanbul, Turkey

**Keywords:** Adolescents, Adults, Children, Congenital heart disease, Heart, Magnetic resonance imaging, Pulmonary valve replacement, Feature tracking strain imaging, Tetralogy of Fallot

## Abstract

**Purpose:**

In patients with repaired tetralogy of Fallot, transcatheter or surgical pulmonary valve replacement is recommended. However, it is not clear whether pulmonary valve replacement preserves systolic and diastolic functions of both ventricles. The aim of the study is to investigate the impact of transcatheter pulmonary valve replacement on atrial and ventricular myocardial strain changes by feature-tracking cardiac magnetic resonance imaging.

**Materials and methods:**

Cardiac magnetic resonance imaging of 18 patients (median age 14.5 years) with repaired tetralogy of Fallot before and after transcatheter pulmonary valve replacement were retrospectively analyzed. Feature tracking strain for both left and right atria and ventricles was performed. Cardiac magnetic resonance imaging parameters (volume and function) and strain characteristics (atria and ventricles) were compared before and after transcatheter pulmonary valve replacement. The Wilcoxon rank-sum and Spearman correlation test was used.

**Results:**

After pulmonary valve replacement, right ventricular end-diastolic volume, end-systolic volume, and stroke volume decreased, whereas left and right ventricular ejection fractions remained unchanged. Reservoir, conduit and pump strain measurements improved for both left (*P* = 0.003, *P* = 0.001, and *P* = 0.006) and right atria (*P* = 0.013, *P* = 0.004, and *P* = 0.015). Global left ventricular circumferential, longitudinal, and radial strains improved (*P* = 0.001, *P* = 0.043, and *P* = 0.002, respectively). Right ventricle global circumferential strain significantly improved with no significant change in the longitudinal and radial strains (*P* = 0.007, *P* = 0.068, and *P* = 0.055, respectively).

**Conclusion:**

Transcatheter pulmonary valve replacement significantly enhances atrial and ventricular strain parameters, indicating a positive impact on overall myocardial function. Feature-tracking cardiac magnetic resonance imaging may offer a comprehensive, non-invasive evaluation of myocardial strain changes in patients with repaired tetralogy of Fallot after pulmonary valve replacement, which leads to improvement of indications and outcomes.

## Introduction

Surgical correction of tetralogy of Fallot often results in pulmonary regurgitation, leading to ventricular dilatation, systolic dysfunction, atrial enlargement, and adverse clinical outcomes such as right heart failure, arrhythmias, and sudden cardiac death [1–3]. Thus, pulmonary valve replacement, either surgical or transcatheter, is recommended in patients with pulmonary regurgitation and/or right ventricular outflow stenosis under specific conditions [1–3]. However, the impact of pulmonary valve replacement on long-term adverse clinical outcomes and reverse atrial and ventricular remodeling is still scarce and controversial [4, 5]. Demonstrating early systolic and diastolic dysfunction by myocardial strain assessment that defines the deformation of fibers through the cardiac cycle before the impairment of ejection fraction is crucial for comprehensive management and risk stratification [6]. However, the complex geometric structure of the right ventricle and limited acoustic window limit the evaluation of myocardial distortion with conventional echocardiography techniques.

Cardiac magnetic resonance imaging (MRI) is widely accepted as the gold standard imaging technique assessing biventricular volume and function, blood flow, and pulmonary regurgitation. It plays a crucial role in clinical decision-making regarding the timing of pulmonary valve replacement, whether surgical or transcatheter, in line with current guidelines [2, 3]. The main advantage of the technique over transthoracic echocardiography is providing increased spatial resolution with a large field of view for the myocardial wall of both ventricles and atria [7]. Recent advancements in non-invasive imaging techniques have also facilitated the direct assessment of atrial and ventricular deformation by feature-tracking strain through cardiac MRI [7–9].

The atrial strain offers insights into atrial function across various phases of the cardiac cycle. *Reservoir function* in systole reflects atrial compliance; passive *conduit function* in early diastole indicates ventricular relaxation and chamber stiffness; and *booster function* represents intrinsic atrial contractility and ventricular compliance through atrial contraction in late diastole [8–11]. It has been of significant importance in assessing atrial remodeling response to pulmonary valve replacement in patients with repaired tetralogy of Fallot in recent years. To the best of our knowledge, only one previous study has evaluated the atrial strain changes by cardiac MRI after transcatheter pulmonary valve replacement [12].

In addition to atrial strain, the evaluation of ventricular strain is considered a more sensitive indicator of ventricular function than volumetric parameters [7]. Although the left and right ventricular strain were significantly associated with adverse outcomes [13, 14], the improvement in global right ventricle strain following pulmonary valve replacement in patients with repaired tetralogy of Fallot remains poorly defined [4, 5].

A better understanding of the effects of transcatheter pulmonary valve replacement on atrial and ventricular function and mechanism may lead to improved indications and outcomes for the procedure. Therefore, our study aimed to evaluate the atrial and ventricular reverse remodeling after transcatheter pulmonary valve replacement through a feature-tracking strain technique by cardiac MRI.

## Materials and methods

Ethical approval of this retrospective study was obtained from the Ethical Committee of the Acıbadem University. Written informed consent was waived for this retrospective study.

### Patient groups and data collection

**Fig. 1:**
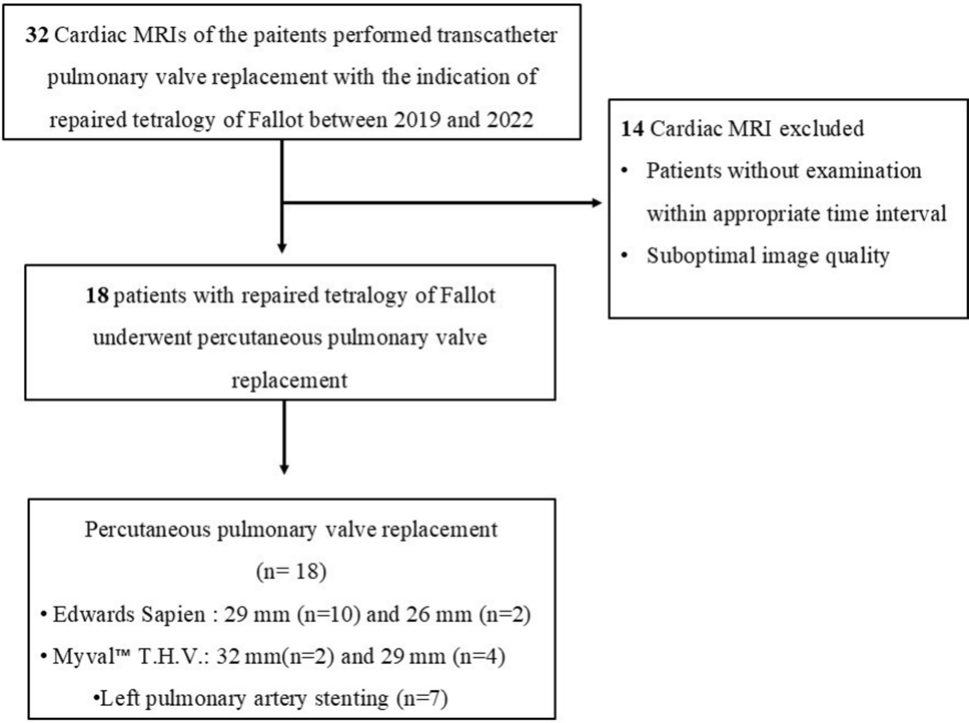
The flowchart of the study cohort

We retrospectively reviewed the cardiac MRI of 32 patients with repaired tetralogy of Fallot who performed transcatheter PVR between 2019 and 2022. The patients who underwent cardiac MRI within one year before and after transcatheter pulmonary valve replacement were included. A total of 18 patients who underwent transcatheter pulmonary valve replacement were included in the study. All patients had the same surgical history of ventricular septal defect closure and the right ventricular outflow tract patch augmentation. Patients with the right ventricle-to-pulmonary artery conduit and valve-in-valve implants were excluded. Demographic data (gender, age) and clinical information (type of surgical procedures, echocardiography, electrocardiography, and cardiac catheterization findings) of the patients were collected from the hospital database system and recorded. The weight and height of the patients were recorded to calculate body surface area for indexed values. The flowchart of the study cohort is presented in Fig. 1.

The indications of transcatheter pulmonary valve replacement were moderate-to-severe pulmonary regurgitation (regurgitation fraction by cardiac MRI ≥ 25%) and at least 2 of the following conditions [1]: right ventricle end-diastolic volume index ≥ 150 ml/m^2^, right ventricle end-systolic volume index ≥ 80 ml/m^2^, right ventricle ejection fraction ≤ 47%, left ventricle end-diastolic volume index ≤ 65 ml/m^2^, severe branch pulmonary artery stenosis (preferential blood flow less than 30% to affected lung) and QRS duration longer than 160 ms on the electrocardiogram. The patients with pulmonary stenosis who had the indication for transcatheter pulmonary valve replacement were excluded. The cardiac MRI examination performed longer than one-year pre-procedure and post-procedure were excluded.

### Cardiac magnetic resonance imaging technique and evaluation

All cardiac MRI images were acquired using a 1.5-Tesla (450W; GE Medical Systems, Waukesha, WI) and 1.5-Tesla (Avanto, Siemens Healthineers, Erlangen, Germany) MRI device with a 32-channel phased-array abdominal coil.

The study protocol included a cardiac MRI study within one year before transcatheter pulmonary valve replacement and within one to two years after pulmonary valve replacement. An ECG-gated balanced steady-state free precession sequence was performed in a two-chamber, four-chamber, right ventricular outflow tract, and short-axis view to assess ventricular volume and function. The scanning parameters for the 450W (General Electric Healthcare) were as follows: TR 3.6 ms; TE 1.5 ms; flip-angle 60°; bandwidth ± 90 kHz; matrix 192 × 192; FOV 350 mm; NEX 1; slice thickness 8 mm; gap 0 mm. The scanning parameters for the Avanto (Siemens Healthineers, Erlangen, Germany) were as follows: repetition time = 3.2 ms, echo time = 1.6 ms, flip angle = 60°, field of view = 275 × 340 mm, matrix size = 224 × 256, slice thickness = 8 mm, slice gap = 0 mm. A phase-contrast imaging sequence was performed of the main pulmonary artery perpendicular to the main pulmonary artery. The optimal velocity encoding value of the pulmonary artery was calculated using the Doppler velocity according to the Bernoulli equation. The applied parameters for the phase contrast imaging sequence for the 450W (General Electric Healthcare) were as follows: TR 5 ms; TE 3 ms; flip-angle 20°; bandwidth ± 60 kHz; matrix 192 × 160; FOV 350 mm; NEX 1; slice thickness 6 mm. The scanning parameters for the Avanto (Siemens Healthineers, Erlangen, Germany) were as follows: TR 31.8 ms; TE 2.9 ms; flip-angle 30°; bandwidth ± 60 kHz; matrix 192 × 160; FOV 350 mm; NEX 1; slice thickness 6 mm. All the examinations were acquired with retrospective electrocardiogram gating and 20–25 reconstructed cardiac phases according to the heart rate. The images were acquired during 14 ± 3 s breath-hold duration, depending on the heart rate during the end-expiratory breath-hold. The duration of the examination changes between 35 and 45 min.

All cardiac MRI examinations were reviewed by a ten-year experienced radiologist (S.O., trained in congenital cardiac imaging with experience of more than 1500 cardiac MRI examinations) without prior knowledge of the clinical details. Biventricular volume, function, strain, and blood flow analyses were performed by using the cardiac imaging software Circle (Circle Cardiovascular Imaging, Calgary, Alberta, Canada). During the assessment of ventricular volume and function, automated contouring was performed by including papillary muscles and the trabeculations as cardiac volume in short-axis images during end-diastole and end-systole for left and right ventricles. Correction of the contouring performance was also performed manually to ensure the accuracy of cardiac MRI parameters (volume, function, and flow analyses). For the assessment of atrial volume, the endocardial contours of the right atrium and left atrium were manually traced in every slice of the stack of four chamber cine images at end-diastole and end-systole. The atrial appendages were included in the measurement of atrial volumes. However, caval veins, coronary sinus, and pulmonary veins were excluded at their junction to the atrium [15]. All volumetric data were indexed and recorded according to the body surface area (using the Mosteller formula). Cine cardiac MRI data was used to evaluate the severity of tricuspid regurgitation in three degrees (mild; first degree, moderate; second degree, severe; third degree). For the flow analysis, the contour of the main pulmonary artery was traced automatically. The pulmonary regurgitation fraction was calculated automatically. The formula is diastolic backward flow/systolic forward flow × 100%. Cine cardiac MRI data were used to evaluate strain analyses. Left and right ventricular strains were computed using the two-chamber, four-chamber, and short-axis cine images, while only the four-chamber cine image was used for left and right atrial strain.

**Fig. 2:**
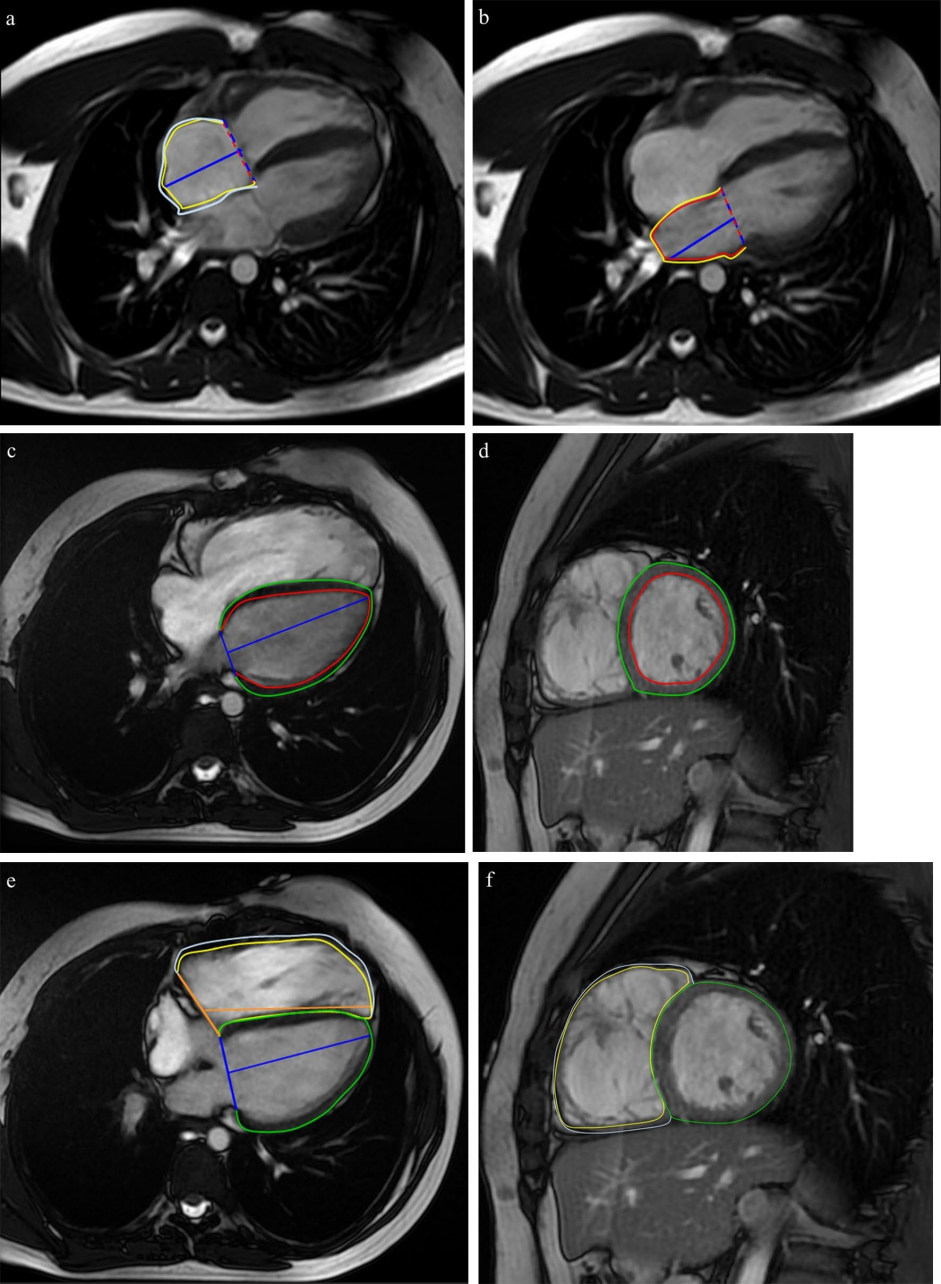
Representative feature-tracking strain cardiac MRI in a16-year-old male with repaired tetralogy of Fallot (**a**–**f**). Atrial strain measurement in four-chamber cine images for right (**a**) and left atria (**b**). Strain measurment of left (**c**, **d**) and right (**e**, **f**) ventricles in four-chamber and short axis cine steady-state free precession images to produce the circumferential longitudinal**,** and radial strain curves

**Fig. 3:**
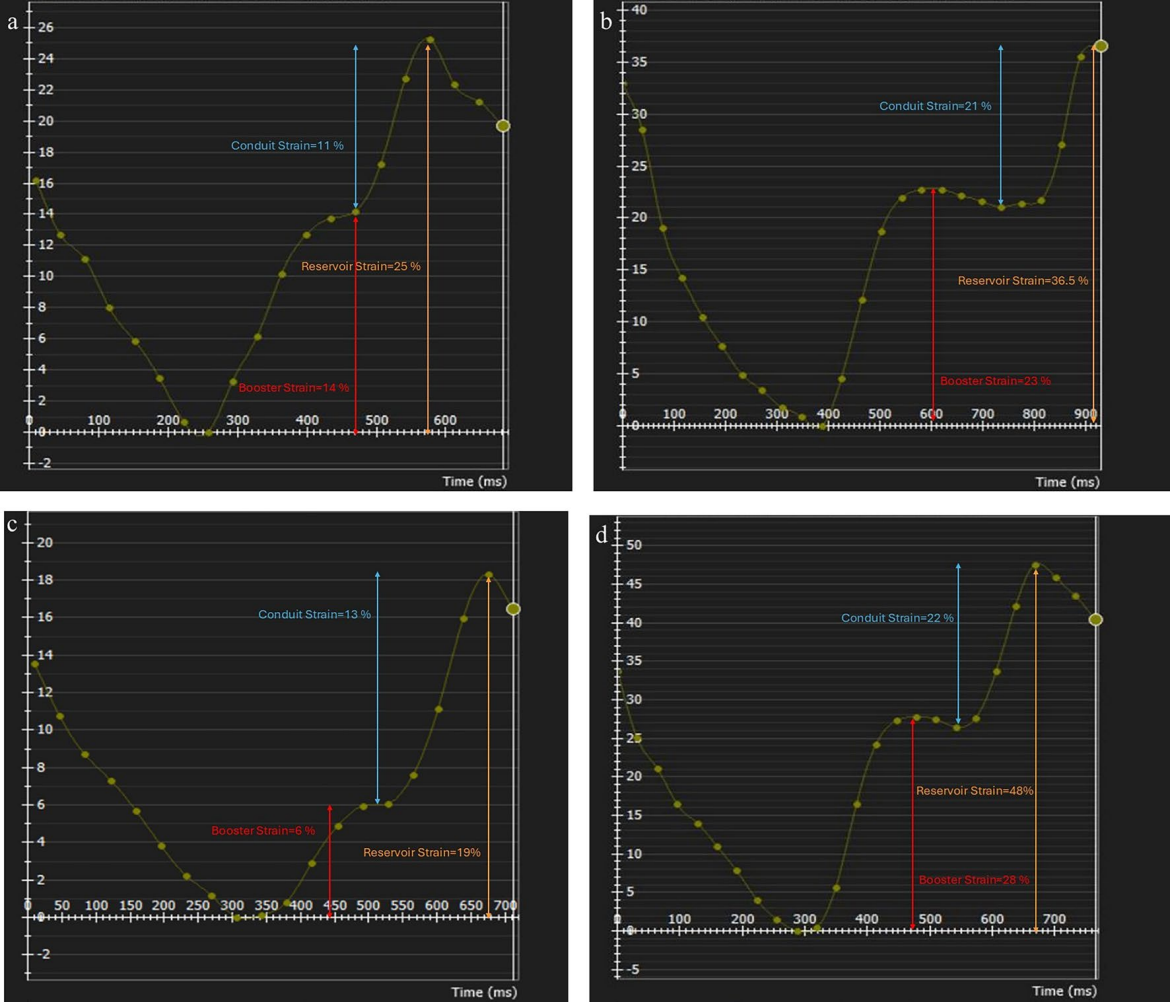
Strain curves of the right and left atria before and after pulmonary valve replacement in a 14-year-old male patient. Strain curves represent the reservoir, conduit, and boost strains for before and after pulmonary valve replacement for rigt atrium (**a**, **b**) and left atrium (**c**, **d**)

For atrial strain assessment, endocardial contours were traced using semi-automatic detection by locating the tricuspid and mitral valves manually. Endocardial and epicardial boundaries at end-diastole and end-systole were traced automatically. The propagated myocardial tissue through ardiac cycle was controlled by the operator, and border contours were corrected as required. Global atrial strain values were automatically derived by the software and controlled. Caval veins, coronary sinus, and pulmonary veins were excluded at their junction to the atrium (Figs. 2, 3). The atrial strain was evaluated in terms of strain parameters as a reservoir (the passive left atrial expansion with blood from the pulmonary veins during left ventricle contraction), conduit (the passive filling of blood from the left atrium to the left ventricle in early-mid left ventricle diastole), and pump strain (the atrial kick in the late, active left ventricle diastole) function [9]. Reservoir and pump strain were determined from the highest and lowest peak on the atrial strain curve. Conduit strain was defined as the difference between the reservoir and pump strain. Reservoir, conduit, and pump strain rates were determined as the first positive systolic peak, earliest negative diastolic peak, and late negative diastolic peak, respectively.

**Fig. 4:**
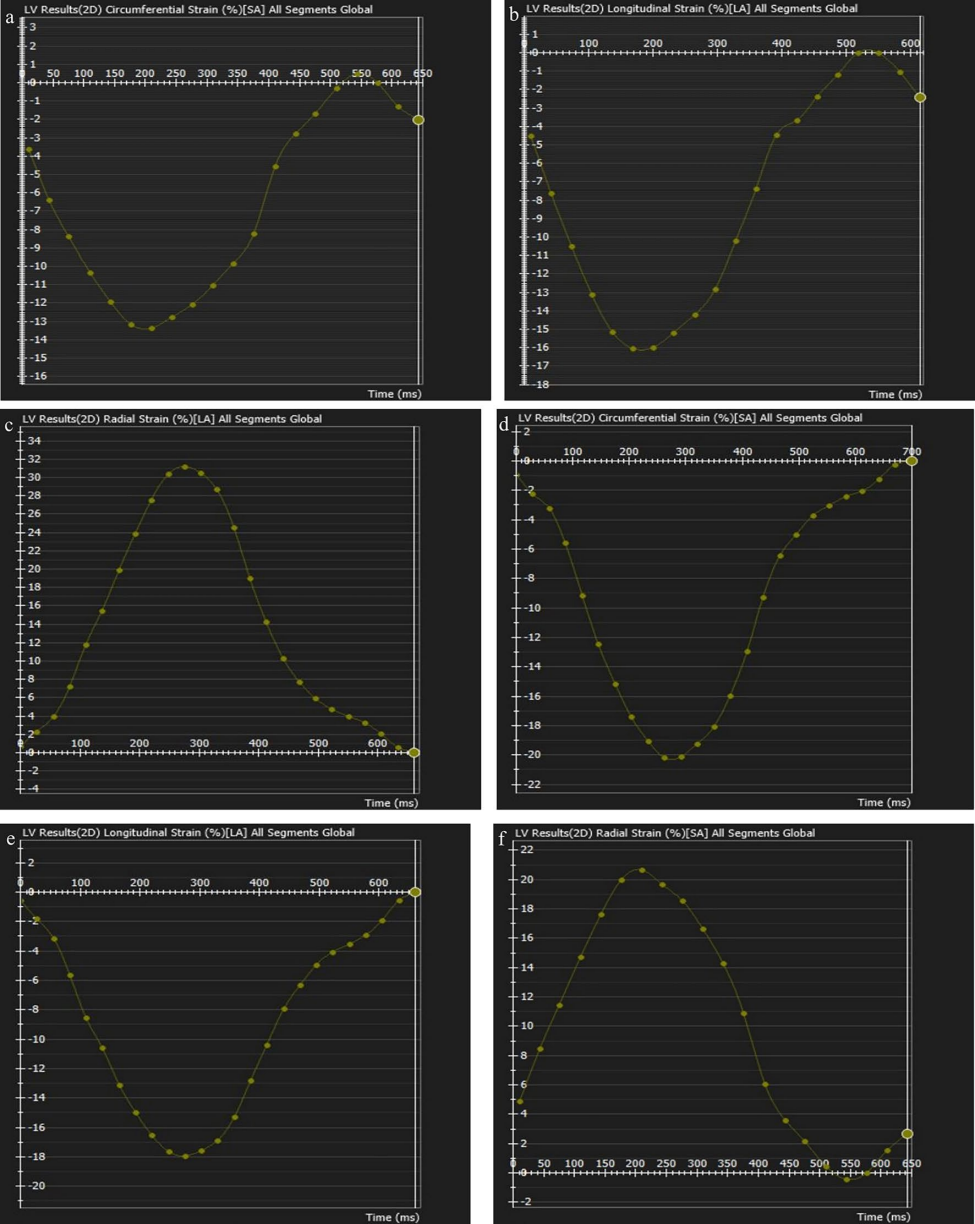
Strain curves of the left ventricle before (**a**–**c**) and after (**d**–**e**) pulmonary valve replacement in a16-year-old male patient. Strain curves represent the circumferential (b,c), longitudinal (**b**,**d**), and radial (**e**,**f**) strain values for left ventricle

**Fig. 5:**
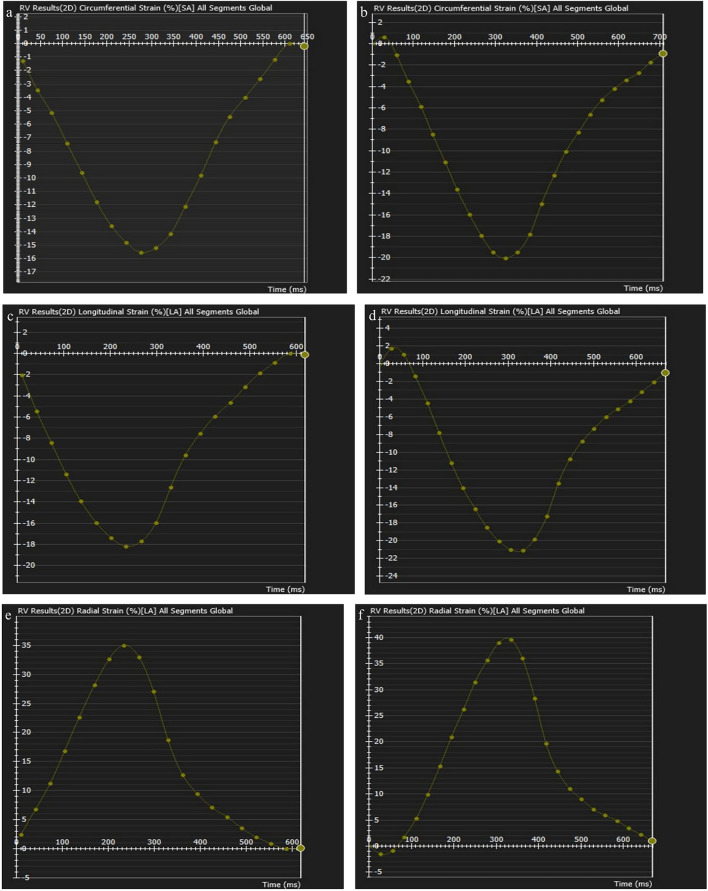
Strain curves of the right ventricle before (**a**–**c**) and after (**d**–**e**) pulmonary valve replacement in a16-year-old male patient. Strain curves represent the circumferential (**b**, **c**), longitudinal (**b**, **d**), and radial (**e**, **f**) strain values for right ventricle

Two and four-chamber cine views were used for global longitudinal ventricular strain analyses, while four-chamber and short-axis cine views were used for global radial and circumferential ventricular strain analyses (Figs. 4, 5). The phase with the largest blood pool area in the middle of the cavity was defined as the end-diastolic phase and the smallest as the end-systolic phase. The endocardium of the ventricles is contoured automatically by the threshold-based segmentation method. The trabeculae and papillary muscles were included in the blood pool. The reference regions at the superior and inferior insertion points of the septum were pointed and drawn manually. The contour of the myocardium for the entire cardiac cycle is propagated automatically by the software. After the contouring was controlled, boundary editing was performed manually, if needed. The software automatically generated the biventricular global radial, circumferential, and longitudinal strain curves. The values of the strain data were recorded.

### Statistical analysis

The analysis of the obtained data was assessed using SPSS version 20.0 software (IBM Corporation, Armonk, NY, USA). The Kolmogorov–Smirnov normality test was applied to evaluate the data distribution. Normally distributed continuous variables are presented as mean ± standard deviation or as median with interquartile range (IQR 25–75) in non-normally distributed data. The Wilcoxon rank-sum test was used to compare the strain and cardiac MRI parameters (volume, function, and pulmonary regurgitation fraction) before and after pulmonary valve replacement. Results with a P value < 0.05 were considered statistically significant. Spearman correlation coefficient was used to analyze simple linear relationships between different variables.

## Results

In total, 18 patients (M/F = 11/7) with repaired tetralogy of Fallot for whom transcatheter pulmonary valve replacement was performed were included in this study. Primary surgery was performed at a median of 18 months of age (interquartile range 12–24 months). All patients had transannular patch augmentation with the closure of the ventricular septal defect. Transcatheter pulmonary valve replacement was performed at a median of 14.5 months of age (interquartile range, 12–16 months). Edwards Sapien^®^ (Edwards Lifesciences, CA, USA) pulmonary valve was used in 12 patients (29 mm in 10 patients and 26 mm in 2 patients), Myval™ T.H.V. (Meril Life Sciences Pvt Ltd, Vapi, Gujarat, India) pulmonary valve in 6 patients (32 mm in 2 patients, 29 mm in 4 patient). Seven patients had left pulmonary artery stenting during transcatheter pulmonary valve replacement (one patient with Myval™ T.H.V. vs. six patients with Edwards Sapien® pulmonary valve). The duration between the first cardiac MRI examination and transcatheter pulmonary valve replacement was 6.5 (interquartile range, 4.75–11.00) months vs. transcatheter PVR, and the second cardiac MRI examination was 11.50 (interquartile range, 9.25–12.00) months. The first cardiac MRI was performed at a median age of, and the mean body surface area was 1.49 (interquartile range, 1.12–1.62) m^2^. The median age of the patients at first and second cardiac MRI was 13.5 (interquartile range, 10.00–15.00) vs. 15.00 (interquartile range, 12.75–16.00) years. Demographic data of the patients are presented in Table 1. The preprocedural QRS was 154.5 (160.5–142.75) msec, and the postprocedural QRS was 155.5 (158.5–143.75) msec (P = 0.074).

**Table 1:** Demographic data of the patients who underwent transcatheter PVR

Parameters	
N (male/female)	18 (11/7)
Age of surgery, months	18.00 (12.00–24.00)
Age of PVR, years	14.5 (12.00–16.00)
Age of first cardiac MRI, years	13.50 (10.00–15.00)
Body surface area at first cardiac MRI, m^2^	1.49 (1.12–1.62)
Age of second cardiac MRI, years	15.00 (12.75–16.00)
Body surface area at second cardiac MRI, m^2^	1.58 (1.41–1.75)
Duration between first cardiac MRI and PVR (months)	6.50 (4.75–11.00)
Duration between PVR and first cardiac MRI (months)	11.50 (9.25–12.00)

Data are presented as mean ± SD or median [25th–75th interquartile range]

*MRI* magnetic resonance imaging*, PVR* pulmonary valve replacement

### Ventricular volume, function, and pulmonary regurgitation fraction

In our study population, right ventricle parameters, including right ventricle end-diastolic volume index (166.50 mL/m^2^ vs. 115.00 mL/m^2^, *P* = 0.000), right ventricle end-systolic volume index (93.00 mL/m^2^ vs. 65.50 mL/m^2^, *P* = 0.000), and right ventricle stroke volume index (67.50 mL/m^2^ vs. 51.00 mL/m^2^, *P* = 0.001), showed significant improvement. There was no significant difference in the right ventricular ejection fraction (41.50% vs. 43.50%, *P* = 0.076). A significant decrease was observed only in left ventricle end-systolic volume index (46.00 mL/m^2^ vs. 41.00 mL/m^2^, *P* = 0.017), while no difference was observed in left ventricle end-diastolic volume index (88.00 mL/m^2^ vs. 82.00 mL/m^2^, *P* = 0.117), left ventricle stroke volume index (42.00 mL/m^2^ vs. 39.50 mL/m^2^, *P* = 0.341), left ventricle ejection fraction (49.50% vs. 51.00%, *P* = 0.074). As expected, the pulmonary regurgitation fraction showed significant improvement (45.50% vs. 2.50%, *P* = 0.000).

### Atrial size, tricuspid regurgitation, and QRS time duration

After transcatheter pulmonary valve replacement, a statistically significant decrease was found in right atrial end-diastolic volume index (58.00 mL/m^2^ vs. 53.50 mL/m^2^, *P* = 0.000), right atrial end-systolic volume index (37.00 mL/m^2^ vs. 34.50 mL/m^2^, *P* = 0.002). However, no significant progression was found in the left atrial end-systolic volume (21.50 mL/m^2^ vs. 21.00 mL/m^2^, *P* = 0.652), and end-diastolic volume index (39.00 mL/m^2^ vs. 37.00 mL/m^2^, *P* = 0.099).

In terms of tricuspid regurgitation, mild tricuspid regurgitation in 7 patients (39%), moderate in 7 patients (39%), and severe in 4 patients (22%) were detected before transcatheter pulmonary valve replacement. After transcatheter pulmonary valve replacement, mild tricuspid regurgitation was detected in 12 patients (67%), moderate in 5 patients (28%), and severe in 1 patient (5%). The severity of tricuspid regurgitation decreased significantly after transcatheter pulmonary valve replacement (P < 0.005). We found no statistically significant change after transcatheter pulmonary valve replacement (155.5 (158.5–143.75) vs. 154.5 (160.5–142.75), respectively, *P* = 0.074). Table 2 demonstrates the cardiac MRI parameters before and after transcatheter pulmonary valve replacement.

**Table 2:** Comparison of cardiac MRI parameters before and after transcatheter PVR in patients with repaired tetralogy of Fallot

	Before PVR (n = 18)	After PVR (n = 18)	*P*	Difference (%)
Right ventricle cardiac MRI parameters				
EDVI, mL/m^2^	166.50 (143.50–184.00)	115.00 (104.50–125.25)	0.000*	30.72
ESVI, mL/m^2^	93.00 (81.50–113.75)	65.50 (58.25–69.25)	0.000*	29.56
SVI, mL/m^2^	67.50 (57.75–71.25)	51.00 (46.75–56.25)	0.001*	24.44
EF, %	41.50 (38.75–44.25)	43.50 (40.00–45.25)	0.076	4.81
Left ventricle cardiac MRI parameters				
EDVI, mL/m^2^	88.00 (81.25–97.25)	82.00 (73.00–92.25)	0.117	6.81
ESVI, mL/m^2^	46.00 (40.00–50.25)	41.00 (35.75–47.25)	0.017*	10.86
SVI, mL/m^2^	42.00 (39.25–51.25)	39.50 (36.75–47.50)	0.341	5.05
EF, %	49.50 (46.5–52.25)	51.00 (48.00–54.25)	0.074	2.94
PR fraction, %	45.50 (38.00–52.00)	2.50 (1.00–21.00)	0.000*	94.50
Other parameters				
Right atrial EDVI	58.00 (55.00–60.75)	53.50 (48.00–55.00)	0.000*	7.75
Right atrial ESVI	37.00 (33.25–42.00)	34.50(28.00–39.00)	0.002*	6.75
Left atrial EDVI	39.00 (34.00–44.00)	37.00 (31.25–40.50)	0.099*	5.12
Left atrial ESVI	21.50 (20.00–22.00)	21.00 (20.25–22.00)	0.652	2.32
TR, n mild/moderate/severe	7/7/4	12/5/1	0.005*	NA
QRS, msec	155.5 (158.5–143.75)	154.5 (160.5–142.75)	0.074	6

Data are presented as median [25th–75th interquartile range]. *P*, for the Wilcoxen-Rank test. *P*-value is significant if < 0.05

*EDVI* end-diastolic volume index, *ESVI* end-systolic volume index, *EF* ejection fraction, *MRI* magnetic resonance imaging*, PVR* pulmonary valve replacement, *PR* pulmonary regurgitation, *SVI* stroke volume index, *TR* tricuspid regurgitation

### Atrial and ventricular strain measurements

There was a significant difference in terms of left atrial reservoir (26.09% vs. 32.20%, *P* = 0.003), conduit (9.50% vs. 13.70%, *P* = 0.001), and pump (17.05% vs. 18.90%, *P* = 0.006) strains after pulmonary valve replacement. There were no differences in the left atrial reservoir (0.95 vs. 0.90; *P* = 0.512), conduit (− 1.25 vs. − 1.30; *P* = 0.932), and pump (− 0.75 vs. − 0.75, *P* = 0.477) strain rate parameters. In terms of right atrial strain, reservoir (21.50% vs. 28.2%, *P* = 0.013), conduit (6.70% vs 9.80%, *P* = 0.004), and pump (14.90% vs. 19.20%, *P* = 0.015) strain values showed a significant improvement after pulmonary valve replacement. Furthermore, there were no differences in the right atrial reservoir (0.90 vs. 1.05; *P* = 0.512), conduit (− 0.90 vs. − 1.10; *P* = 0.125), and pump (− 1.10 vs. − 1.10, *P* = 0.484) strain rates. Table 3 shows the atrial strain parameters in the patients before and after pulmonary valve replacement.

**Table 3:** Comparison of atrial strain cardiac MRI parameters before and after transcatheter PVR in patients with repaired tetralogy of Fallot

	Before PVR (n = 18)	After PVR (n = 18)	*P*	Difference (%)
Left atrıal global longıtudınal straın parameters				
Reservoir (%)	26.09 (20.60–29.77)	32.20 (30.00–48.90)	0.003*	9.92
Conduit (%)	9.50 (7.20–11.57)	13.70 (11.35–23.70)	0.001*	44.21
Pump (%)	17.05 (12.52–18.67)	18.90 (17.80–24.25)	0.006*	10.85
Rıght atrıal global longıtudınal straın parameters				
Reservoir (%)	21.50 (17.10–24.05)	28.2 (25.80–32.10)	0.013*	12.2
Conduit (%)	6.70 (3.80–9.05)	9.80 (8.60–14.75)	0.004*	46.2
Pump (%)	14.90 (10.90–18.25)	19.20 (14.20–20.70)	0.015*	28.85
Left atrıal strain rate parameters				
Reservoir strain rate (s^−1^)	0.95 (0.67–0.155)	0.90 (070–1.00)	0.512	5.26
Conduit strain rate (s^−1^)	− 1.25 (− 1.75 to − 1.00)	− 1.30 (− 1.8 to − 1.20)	0.932	4
Pump strain rate (s^−1^)	− 0.75 (− 1.13 to − 0.57)	− 0.75 (− 1.10 to − 0.63)	0.477	NA
Rıght atrıal strain rate parameters				
Reservoir strain rate (s^−1^)	0.90 (0.65–1.15)	1.05 (0.80–1.35)	0.439	16.6
Conduit strain rate (s^−1^)	− 0.90 (− 1.25 to − 0.50)	− 1.10 (− 1.48 to − 0.83)	0.125	22.2
Pump strain rate (s^−1^)	− 1.10 (− 1.25 to − 0.75)	− 1.10 (− 1.40 to − 0.95)	0.484	NA

Data are presented as median [25th–75th interquartile range]. *P*, for the Wilcoxen-Rank test. *P*-value is significant if < 0.05

*MRI* magnetic resonance imaging*, PVR* pulmonary valve replacement

There was a significant improvement in global left ventricular circumferential (− 15.80% vs*.* − 19.45%,* P* = 0.001), longitudinal (− 17.75% vs. − 18.80%, *P* = 0.043), and radial (26.85% vs. 33.05%, *P* = 0.002) strain parameters after pulmonary valve replacement. A significant improvement in right ventricular global circumferential strain was revealed after transcatheter pulmonary valve replacement (− 14.25% vs. − 16.30%, *P* = 0.007). Furthermore, right ventricular global longitudinal (− 18.05% vs. − 18.50%,* P* = 0.068) and radial (32.00% vs. 32.95%, *P* = 0.055) strain parameters were unchanged after pulmonary valve replacement. Table 4 shows the global strain parameters for the left and right ventricles before and after pulmonary valve replacement.

**Table 4:** Comparison of ventricular strain cardiac MRI parameters before and after transcatheter PVR in patients with repaired tetralogy of Fallot

	Before PVR (n = 18)	After PVR (n = 18)	*P*		Difference (%)	
Left ventrıcle global straın parameters						
Circumferential, %	− 15.80 (− 17.65 to − 14.22)	− 19.45 (− 20.20 to − 18.32)	0.001*		23.1	
Longitudinal, %	− 17.50 (− 19.20 to − 12.93)	− 18.80 (− 19.45 to − 16.23)	0.043*		7.4	
Radial, %	26.85 (22.53–30.00)	33.05 (30.60–36.03)	0.002*		24.4	
Rıght ventrıcle global straın parameters						
Circumferential, %	− 14.25 (− 16.42 to − 12.75)	− 16.30 (− 18.52 to − 14.82)		0.007*		14.4
Longitudinal, %	− 18.05 (− 18.85 to − 16.17)	− 18.50 (− 20.72 to − 17.82)		0.068		NA
Radial, %	32.00 (24.53–34.60)	32.95 (30.78–40.58)		0.055		2.9

Data are presented as median [25th–75th interquartile range]. *P*, for the Wilcoxen-Rank test. *P*-value is significant if < 0.05

*MRI* magnetic resonance imaging*, PVR* pulmonary valve replacement

For the right atrial strain, in the *reservoir phase*, there were weak negative correlations between right ventricular end-systolic volume index (P = 0.911, r =  − 0.029), pulmonary regurgitation (P = 0.925, r =  − 0.025), and tricuspid regurgitation (P = 0.881, r =  − 0.039) with right atrial strain, without statistical significance difference. There was a weak positive correlation between right ventricular end-diastolic volume index (P = 0.779, r = 0.074), right ventricular ejection fraction (P = 0.891, r = 0.036), and atrial strain, which was not statistically significant. In the *conduit phase*, there was a moderate negative correlation between right ventricular end-diastolic volume index (P = 0.149, r =  − 0.365), right ventricular end-systolic volume index (P = 0.715, r =  − 0.096), pulmonary regurgitation (P = 0.963, r =  − 0.012), and tricuspid regurgitation (P = 0.172, r =  − 0.347) with right atrial strain, however this also was not statistically significant. There was a weak positive correlation between right ventricular ejection fraction (P = 0.721, r = 0.094) and atrial strain, which was not statistically significant. In the *pump phase*, there was a weak negative relationship between right ventricular end-diastolic volume index (P = 0.428, r =  − 0.206), tricuspid regurgitation (P = 0.336, r =  − 0.249), and right atrial strain which was not significant. There was a weak positive correlation between right ventricular end-systolic volume index (P = 0.925, r = 0.025), right ventricular ejection fraction (P = 0.175, r = 0.345), Pulmonary regurgitation (P = 0.981, r = 0.006) and atrial strain, which was not statistically significant. We found no statistically significant correlation between QRS duration and atrial strain change. The correlation between right and left atrial strain chances and cardiac MRI parameters are also presented in Table 5.

**Table 5:** Correlation between pre-procedure end-diastolic volume index, pulmonary regurgitation fraction, and atrial strain MRI parameters

	Rıght atrıum straın			Left atrıum straın		
	Reservoir	Conduit	Pump	Reservoir	Conduit	Pump
RVEDVI						
P	0.779	0.149	0.428	0.141	0.859	0.341
r	0.074	− 0.365	− 0.206	0.361	0.047	− 0.246
RVESVI						
P	0.911	0.715	0.925	0.083	0.981	0.322
r	− 0.029	− 0.096	0.025	0.421	0.006	− 0.256
RVEF						
P	0.891	0.721	0.175	0.702	0.286	0.616
r	0.036	0.094	0.345	0.097	0.274	0.131
PR						
P	0.925	0.963	0.981	0.165	0.757	0.342
r	− 0.025	− 0.012	0.006	0.342	0.081	− 0.246
TR						
P	0.881	0.172	0.336	0.236	0.544	0.475
r	− 0.039	− 0.347	− 0.249	0.294	0.158	− 0.189
QRS						
P	0.247	0.214	0.185	0.133	0.599	0.354
r	0.297	− 0.318	− 0.338	0.380	− 0.138	− 0.240

*P*, for the Spearman Correlation Test test. R for correlation coefficient value. *P*-value is significant if < 0.05

*MRI* magnetic resonance imaging*, PVR* pulmonary valve replacement, *PR* pulmonary regurgitation, *RVEDVI* right ventricle end-diastolic volume index, *RVESVI* right ventricle end-systolic volume index, *RVEF* right ventricle ejection fraction, *TR* tricuspid regurgitation

For the right ventricular strain, in the *circumferential strain*, there was a negative correlation between right ventricular end-diastolic volume index (P = 0.686, r =  − 0.102), right ventricular end-systolic volume index (P = 0.449, r =  − 0.171), pulmonary regurgitation (P = 0.182, r = 0.329) and with tricuspid regurgitation (P = 0.997, r =  − 0.001), which were not statistically significant. In the *longitudinal strain*, there was a negative correlation between right ventricular end-diastolic volume index (P = 0.804, r =  − 0.063), right ventricular end-systolic volume index (P = 0.447, r =  − 0.191), pulmonary regurgitation (P = 0.184, r = 0.331) and with tricuspid regurgitation (P = 0.611, r =  − 0.129), which were not statistically significant. In the *radial strain*, a significant negative correlation was found between pulmonary regurgitation and right ventricular radial strain (r =  − 0.541, P = 0.021). There was a negative correlation between right ventricular end-diastolic volume index (P = 0.766, r =  − 0.075), right ventricular end-systolic volume index (P = 0.114, r =  − 0.386), and with tricuspid regurgitation (P = 0.555, r =  − 0.149), which were not statistically significant. There was a positive correlation between right ventricular ejection fraction and *circumferential strain* (P = 0.455, r = 0.581), *longitudinal strain* (P = 0.395, r = 0.213), *radial strain* (P = 0.305, r = 0.256), which were not statistically significant.

For the left ventricular strain, in the *circumferential strain*, a significant negative correlation was found between the right ventricular end-diastolic volume index (P = 0.037, r =  − 0.494). There was a negative correlation between right ventricular end-diastolic volume index (P = 0.086, r =  − 0.416), pulmonary regurgitation (P = 0.747, r =  − 0.082), tricuspid regurgitation (P = 0.248, r =  − 0.287), and a positive correlation with right ventricular ejection fraction (P = 0.758, r = 0.078), which were not statistically significant. In the *longitudinal strain*, there was a negative correlation between right ventricular end-diastolic volume index (P = 0.108, r =  − 0.391), right ventricular end-systolic volume index (P = 0.255, r =  − 0.283), pulmonary regurgitation (P = 0.848, r =  − 0.089), tricuspid regurgitation (P = 0.379, r =  − 0.221), and a positive correlation with right ventricular ejection fraction (P = 0.119, r = 0.848), which were not statistically significant. In the *radial strain*, there was a positive correlation between right ventricular end-diastolic volume index (P = 0.089, r = 0.412), right ventricular end-systolic volume index (P = 0.176, r = 0.334), right ventricular ejection fraction (P = 0.876, r = 0.040), pulmonary regurgitation (P = 0.701, r = 0.097), and tricuspid regurgitation (P = 0.329, r = 0.242), which were not statistically significant. We found no statistically significant correlation between QRS duration and ventricular strain change.The correlation between right and left ventricular strain chances and cardiac MRI parameters are also presented in Table 6.

**Table 6:** Correlation between pre-procedure end-diastolic volume index, pulmonary regurgitation fraction, and ventricular strain MRI parameters

	Rıght ventrıcle straın			Left ventrıcle straın		
	Circumferential	Longitudinal	Radial	Circumferential	Longitudinal	Radial
RVEDVI						
P	0.686	0.804	0.766	0.086	0.108	0.089
r	− 0.102	− 0.063	− 0.075	− 0.416	− 0.391	0.412
RVESVI						
P	0.497	0.447	0.114	0.037*	0.255	0.176
r	− 0.171	− 0.191	− 0.386	− 0.494	− 0.283	0.334
RVEF						
P	0.455	0.395	0.305	0.758	0.639	0.876
r	0.581	0.213	0.256	0.078	0.119	0.040
PR						
P	0.182	0.184	0.021*	0.747	0.848	0.701
r	− 0.329	− 0.331	− 0.541	− 0.082	− 0.089	0.097
TR						
P	0.997	0.611	0.555	0.248	0.379	0.329
r	− 0.001	− 0.129	− 0.149	− 0.287	− 0.221	0.242
QRS						
P	0.636	0.527	− 0.049	− 0.310	0.445	0.334
r	− 0.119	− 0.159	0.844	0.209	− 0.192	0.175

*P* for the Spearman correlation test. R for correlation coefficient value. *P*-value is significant if < 0.05

*MRI* magnetic resonance imaging*, PVR* pulmonary valve replacement, *PR* pulmonary regurgitation, *RVEDVI* right ventricle end-diastolic volume index, *RVESVI* right ventricle end-systolic volume index, *RVEF* right ventricle ejection fraction, *TR* tricuspid regurgitation

## Discussion

In this study, we evaluated atrial and ventricular reverse remodeling by feature-tracking cardiac MRI strain technique after transcatheter pulmonary valve replacement in patients with repaired tetralogy of Fallot. The main findings of this study are (1) a significant increase in global longitudinal strain parameters (conduit, pump, and reservoir) for left and right atria, (2) a significant increment in the global circumferential strain parameters of the right ventricle, (3) a significant improvement of global circumferential, longitudinal, and radial left ventricle strain parameters, and (4) a significant correlation between left ventricular circumferential strain change and right ventricular end-systolic volume, and (5) a significant improvement of the right ventricle function and cardiac MRI parameters one year after transcatheter pulmonary valve replacement.

It has been of significant importance in assessing atrial remodeling response to pulmonary valve replacement in patients with repaired tetralogy of Fallot in recent years. In a study by Abozied OA et al., it was revealed that the preoperative right atrial reservoir strain was associated with right atrial reverse remodeling after surgical pulmonary valve replacement [16]. Another study from the same group, Egbe AE et al. showed that the patients who underwent transcatheter pulmonary valve replacement had a significant improvement in the right atrial and ventricular function compared with those who underwent surgical pulmonary valve replacement [17]. However, two studies were designed by echocardiography-based strain assessment. To the best of our knowledge, only one previous study has evaluated the atrial strain changes by cardiac MRI after pulmonary valve replacement [12]. In our study, a significant improvement was found in global longitudinal reservoir, conduit and pump strain parameters for right atria. Our results were similar with the literature [12]. The improvement in conduit and pump strains suggest improved ventricular relaxation and compliance, reflecting reduced ventricular stiffness post-procedure. The stability in reservoir function highlights the atria's maintained capacity to store and release blood effectively, possibly facilitated by the absence of severe tricuspid regurgitation in the cohort. These findings underline the critical role of atrial function in optimizing diastolic filling and overall cardiac performance [16, 17].

Besides the clinical importance of atrial strain assessment, the evaluation of myocardial distortion is accepted to be a more sensitive indicator of ventricular function than volumetric parameters [8]. In our study, we also observed higher global circumferential, longitudinal, and radial strain of the left ventricle. The global circumferential strain of the right ventricle showed significant improvement following transcatheter pulmonary valve replacement. In contrast, the right ventricular global longitudinal and radial strain parameters remained unchanged. In a study by Balasubramanian S. et al., a significant improvement was found in left ventricle circumferential strain in the mid (mean of 7 months following operation) post-operative period by cardiac MRI. Nevertheless, no significant change was revealed in right ventricle circumferential, longitudinal strain, and left ventricle longitudinal strain parameters [8]. Another study by Kawakubo et al. also found similar results with no differences in global circumferential and longitudinal strain parameters of the right ventricle before and after pulmonary valve replacement [18]. On the other hand, Sjöberg P. et al. revealed that patients before surgical pulmonary valve replacement had lower right ventricle free wall longitudinal strain after pulmonary valve replacement. There was no significant difference in left ventricle global longitudinal strain after pulmonary valve replacement [19]. These results would be associated with the pulmonary valve replacement technique, which was performed using a surgical technique requiring thoracotomy and right ventricle outflow tract transection in both studies. The left ventricle is anatomically and functionally interdependent with the right ventricle. Improvements in right ventricular function following transcatheter pulmonary valve replacement can lead to enhanced left ventricular filling and function due to better diastolic interactions between the two chambers [20]. This interdependence often results in more pronounced improvements in left ventricle strain metrics compared to right ventricle strains post-intervention. On the other hand, the left ventricle typically has a greater reserve capacity for adaptation compared to the right ventricle. After transcatheter pulmonary valve replacement, the reduction of pressure overload on the left ventricle allows it to recover from previous maladaptive changes due to chronic volume overload from pulmonary regurgitation [21, 22]. This recovery process can lead to significant enhancements in global longitudinal and circumferential strains of the left ventricle. Although the patients in our study group are asymptomatic, the patient-specific factors may contribute to variability in right ventricular recovery and response to transcatheter pulmonary valve replacement. In a study by Mauger CA et al. provides insights into how increased pulmonary regurgitant volume in patients with repaired tetralogy of Fallot is associated with distinct biventricular structural and functional changes [23]. It is suggested that higher pulmonary regurgitant volume is correlated with regional right ventricle dilation, basal bulging, and increased right ventricle strain, alongside left ventricle flattening and altered septal motion toward the right ventricle in systole. These findings suggest that right ventricle volume overload impacts not only right ventricle morphology but also has repercussions on left ventricle shape and function, likely due to ventricular interdependence [23, 24]. Understanding these individual characteristics is essential for tailoring follow-up and therapeutic strategies to optimize right ventricular function and long-term outcomes in asymptomatic patients after transcatheter pulmonary valve replacement.

The lack of improvement in right ventricular global longitudinal and radial strain parameters compared to left ventricular indices may be due to inadequate restoration of normal volume and loading conditions with transcatheter pulmonary valve replacement after irreversible myocardial injury after the initial correction surgery. In another study, after initial corrective surgery, both left ventricular and right ventricular systolic and diastolic functions were found to be impaired, with more noticeable deterioration in the right ventricle [25]. In the literature, no definitive explanation has been provided for the postoperative changes after initial corrective surgery. Contributing factors include local tissue damage to the thin-walled, anteriorly positioned right ventricle, effects of cardiopulmonary bypass with limited right ventricle protection by cold cardioplegia, and issues related to pericardiotomy or pericardial adhesions [26]. Although, the patients often present with increased right ventricle pressure and/or volume overload preoperatively, right ventricular deterioration is shown to persist in the medium-term follow-up, and continues to decline further during long-term follow-up [27, 28].

In our study, the negative correlation between right ventricle end-systolic volume and left ventricular circumferential strain would be explained as* ‘volume overload effects’,* which means elevated right ventricle volume often leads to increased pressure and volume load on the left ventricle due to the shared physiology of the heart [29, 30]. Although our patient group had no increased pulmonary artery stenosis and/or increased pulmonary artery gradient, transcatheter pulmonary valve replacement alleviates volume overload, it allows for improved left ventricle function and strain parameter [29]. Also, there is a reduction in right ventricle volume after transcatheter pulmonary valve replacement, which would be explained by ‘reverse remodeling’ affects the left ventricle, enhancing its myocardial performance [31]. This remodeling process can lead to improved contractility and strain metrics. Although there is a non-significant correlation, our study indicated that patients with higher preoperative pulmonary regurgitation showed more significant improvements in left ventricle strain after transcatheter pulmonary valve replacement. High pulmonary regurgitation contributes to right ventricle dilation and impaired function, adversely affecting left ventricle filling and performance. By reducing pulmonary regurgitation through valve replacement, the hemodynamic burden on the left ventricle is relieved, leading to enhanced strain measurements [1, 32]. On the other hand, our study indicated that patients with higher preoperative pulmonary regurgitation fraction showed greater improvements in right ventricle radial strain after transcatheter pulmonary valve replacement [33, 34]. Patients with severe pulmonary regurgitation or high right ventricle end-systolic volume may benefit most from intervention, as they are likely to experience substantial improvements in left ventricle function.

In recent years, transcatheter pulmonary valve replacement has been an alternative technique in selected patients without right ventricular outflow tract aneurysm or other indications for surgical correction [35]. However, limited studies focusing on the effect of transcatheter pulmonary valve replacement on ventricular strain have been designed on the variable characteristics of right ventricular outflow tract morphology, and initial surgical history is often based on echocardiography-based techniques [4, 36–38]. In a study by Irwin M. et al., no significant difference was demonstrated regarding right and left ventricle longitudinal and circumferential strain through cardiac MRI after pulmonary valve replacement. Notably, pulmonary valve replacement was performed dominantly surgically (almost 58% of the cohort) [39]. The unchanged or worsened right ventricular function in various studies might be attributed to the surgical pulmonary valve replacement technique requiring thoracotomy and right ventricle outflow tract transection [7, 18, 19]. Surgical pulmonary valve replacement, while a standard practice for many years, requires extracorporeal circulation, sternotomy, and right ventricular outflow tract dissection. On the other hand, a study from our group evaluated the strain characteristic of the repaired tetralogy of Fallot patients before pulmonary valve implantation and reported that the patients referred to surgical technique had worse ventricular strain parameters than the transcatheter group [40]. The lack of improvement in right ventricle strain parameters after the surgical pulmonary valve replacement group in the literature would be explained by worse baseline strain parameters before the procedure and the invasive nature of the technique. Similarly, a better response of the left ventricle than the right ventricle may prove, considering that the right ventricle is subject to complications from pulmonary valve disease and multiple surgical procedures [13, 41]. The underlying initial anatomy and surgery-related factors may have had unfavorable effects on normal cardiac remodeling.

A significant decrease in right ventricle volume without improvement in the right ventricle ejection fraction was found in our study, as expected. Based on the effects of pulmonary valve replacement, previous studies revealed similar results in terms of ventricular volume and function [4, 42, 43]. Balasubramanian S. et al. found that right ventricle end-diastolic volume, end-systolic volume, and ejection fraction declined, and left ventricle end-diastolic volume and end-systolic volume both increased after surgical pulmonary valve replacement. However, no significant change was revealed in the left ventricle ejection fraction [7]. The common results in these studies are a decrease in right ventricle end-diastolic volume index, with no change in right ventricle and left ventricle ejection fractions [4, 37, 44].

This study had some limitations that must be highlighted. The retrospective methodology of the research and the small cohorts in the single-center group represents the main limitation. Moreover, considering the potential impact of selection bias on the study results is crucial for interpreting the findings accurately [45]. Several factors may contribute to selection bias as short post-procedure cardiac MRI follow-up duration. However, long follow-up cardiac MRI duration may reflect chronic adaptations rather than immediate post-procedure changes. Although all the patients had augmentation native right ventricle outflow tract with closure of ventricular septal defect, the variability in age of initial surgery and surgical process of referral patient would accepted as a potential impact of selection bias. However, all cardiac MRI examinations were performed using an institutionally approved clinical protocol and evaluated by experts in congenital heart disease. In addition, we did not report inter-study reproducibility. Nevertheless, inter-study reproducibility of strain measured by feature-tracking cardiac MRI has been reported in various studies, showing a satisfactory coefficient of variation and intraclass correlation coefficient [8, 37]. Large-sample multicenter prospective longitudinal studies with appropriate follow-up time would be warranted.

In conclusion, transcatheter pulmonary valve replacement in patients with repaired tetralogy of Fallot leads to significant alterations in atrial and ventricular strain parameters without significant changes in volume and function characteristics. Feature-tracking cardiac MRI strain analyses applied to standard cine images without performing additional time-consuming sequences would be included in the repaired tetralogy of the Fallot imaging protocol.
